# DNA methylation and expression analyses reveal epialleles for the foliar disease resistance genes in peanut (*Arachis hypogaea* L.)

**DOI:** 10.1186/s13104-020-4883-y

**Published:** 2020-01-07

**Authors:** R. S. Bhat, J. Rockey, Kenta Shirasawa, I. S. Tilak, M. P. Brijesh Patil, V. B. Reddy Lachagari

**Affiliations:** 1 Department of Biotechnology, University of Agricultural Sciences, Dharwad, 580 005 India; 2 AgriGenome Labs Pvt. Ltd., Kochi, Kerala 682 042 India; 3 Department of Frontier Research, Kazusa DNA Research Institute, Chiba, 292-0818 Japan; 4 AgriGenome Labs Pvt. Ltd., Hyderabad, Telangana 500 078 India

**Keywords:** Peanut, Genotypes differing for foliar disease response, DNA methylome, Transcriptome, Differentially methylated genes, Expression levels of methylated genes

## Abstract

**Objective:**

Low DNA sequence polymorphism despite enormous phenotypic variations in peanut indicates the possible role of epigenetic variations. An attempt was made to analyze genome-wide DNA methylation pattern and its influence on gene expression across 11 diverse genotypes of peanut.

**Results:**

The genotypes were subjected to bisulfite sequencing after 21 days of sowing (DAS). CHG regions showed the highest (30,537,376) DNA methylation followed by CpG (30,356,066) and CHH (15,993,361) across 11 genotypes. The B sub-genome exhibited higher DNA methylation sites (46,294,063) than the A sub-genome (30,415,166). Overall, the DNA methylation was more frequent in inter-genic regions than in the genic regions. The genes showing altered methylation and expression between the parent (TMV 2) and its EMS-derived mutant (TMV 2-NLM) were identified. Foliar disease resistant genotypes showed significant differential DNA methylation at 766 sites corresponding to 25 genes. Of them, two genes (*Arahy.1XYC2X* on chromosome 01 and *Arahy.00Z2SH* on chromosome 17) coding for senescence-associated protein showed differential expression with resistant genotypes recording higher fragments per kilobase of transcript per million mapped reads (FPKM) at their epialleles. Overall, the study indicated the variation in the DNA methylation pattern among the diverse genotypes of peanut and its influence of gene expression.

## Introduction

Peanut (*Arachis hypogaea* L. 2n = 4x = 40) is an important legume food and oilseed crop world-wide. The breeding efforts mainly focus on productivity, disease and insect resistance, oil quality etc. Genomics-assisted breeding has been successfully employed [1, 2] with the development of the genomic resources including the genome sequence of the cultivated allotetraploid peanut [3, 4]. Apart from the genome, the epigenome also influences the gene function and the phenotype [5]. Despite the narrow genetic base, peanut shows enormous phenotypic variability, which probably hints at the possible role of epigenetic variations in altering the phenotype.

DNA methylation along with histone modifications and chromatin changes generally make up the epigenome. In plants, genome-wide DNA methylation analyses have indicated the modification of epigenome with drought [6], growth and development, hybridization [7, 8], induced and spontaneous mutations [9–15] etc. The pattern of DNA methylation within the genome of Arabidopsis [16], rice [9], wheat [17], maize [18], *Brassica juncea* [6], *Plantago lagopus* [19] etc. has been uncovered, and its interdependency on transcription was demonstrated. The stability and heritability of the epigenetic variations are being characterized [20].

Though the putative genes coding for cytosine-5 DNA methyltransferase (C5-MTases) and demethylase mediating the DNA methylation pattern have been identified in the progenitors of peanut [21], the pattern of DNA methylation in the cultivated peanut is yet to be analyzed. In this study, an effort was made to analyze the DNA methylome and its influence on transcriptome of the peanut genotypes contrasting for the productivity traits and the response to foliar diseases with an objective of identifying the epialleles contributing for the desirable phenotype in peanut so that an efficient breeding programme can be devised for such traits.

## Main text

### Methods

A total of 11 genotypes (Table 1) were employed for methylome sequencing. Seeds of these genotypes were sown in the pots and the seedlings were grown in the greenhouse with optimal conditions. Leaf samples were collected for DNA and RNA isolation from a single 21-day old seedling. DNA was isolated using Qiagen DNeasy Plant Mini Kit (Cat # 69104) and the RNA was isolated using Qiagen RNeasy Plant Mini Kit (Cat # 74904). Bisulfite treatment was done using Zymo EZ DNA Methylation-Gold Kit. DNA methylome library was constructed using illumine TruSeq^®^ DNA Methylation Kit. RNA library was constructed using Illumina TruSeq^®^ Stranded mRNA Kit. The quality of the libraries was checked using TapeStation and Qubit. DNA sequencing was carried out using Illumina Hiseq 2500, and the RNA sequencing was carried out using Illumina Hiseq 2500 and Illumina Hiseq 4000 with two technical replicates and without any biological replicates.

**Table 1:** Genotypes used for DNA methylome and transcriptome sequencing

S. no.	Genotypes	Pedigree	Features
1	GPBD 4	KRG 1 × CS 16 (ICGV 86855)	Early maturing with high pod growth rate, has high oil content and resistant to LLS and rust
2	VG 9514	Derivative of *A. cardenasii* and cv. CO 1	Rust resistant
3	ICGV 86855	Interspecific derivative (A *hypogaea* x *A. cardenasii*)	Resistant to LLS, rust and drought
4	ICGV 86699	Derivative of [*A. hypogaea* × (*A. batizocoi* × *A. duranensis*)]	Multiple resistance to ELS, LLS, rust, bud necrosis, stem and pod rots
5	ICGV 99005	Derivative of [*A. hypogaea *× (*A. batizocoi* × *A. duranensis*)]	Immune or with high resistance to LLS and rust
6	TAG 24	Selection from TGS-2 (TG-18A × M13) × TGE-1	Early maturity, high harvest index (50–55%) and susceptible to LLS and rust
7	TMV 2	Selection from ‘Gudhiyatham bunch’	Early maturity, wide adaptability and susceptible to LLS and rust
8	JL 24	Selection from ‘EC-9493’	Early maturity, high yielding short duration and susceptible to LLS and rust
9	DER	Dh 3-20 × CGC-1	Runner type and susceptible to LLS
10	VL 1	Mutant of DER	Rust resistant
11	TMV 2-NLM	Mutant of TMV 2	Drought tolerant and resistant to LLS and rust

#### Methyl-Seq analysis

Raw fastq files were pre-processed using AdapterRemoval v2 [22] tool. Using bwa-meth [23] program, the preprocessed reads were aligned with the *Arachis hypogaea* reference genome downloaded from PeanutBase [24]. The genomic sites showing DNA methylation were identified using MethylDackel program. Differential methylation was analyzed using methylKit [25] R package. The DNA methylation pattern was compared across the genotypes at *q*-value cutoff 0.01 and methylation percentage change cutoff 25 using methylKit.

#### RNA-Seq analysis

Raw data pre-processing was done using AdapterRemoval v2 [22] tool. The pre-processed reads were aligned with silva database using bowtie2 [26] to remove any ribosomal RNA. The reference genome of the *Arachis hypogaea* was downloaded from PeanutBase [24] and employed for alignment using STAR aligner [27] to get the alignment bam files. Differential expression analysis was executed using cuffdiff program of cufflinks package [28]. log2 fold change cutoff ± 2 and *P*-value cutoff 0.05 were used for identifying differentially expressed genes. Custom scripts were developed to identify genes that were differentially methylated as well as differentially expressed.

#### qRT-PCR

Expression of the selected genes were confirmed using qRT-PCR. Total RNA was isolated from the young leaves on 21 DAS using Qiagen Rneasy mini kit (Qiagen, USA), and the quantity was checked using NanoDrop spectrophotometer (ND-2000, Thermo fisher scientific, USA). cDNA was synthesized using Affinity Script qPCR cDNA synthesis kit (Agilent Technologies, USA). qPCR assay was carried out to check the expression levels of selected genes using SYBR Green chemistry (Brilliant II SYBR Green qPCR master mix (Agilent Technologies, USA) using two technical replicates. Level of fold change over a house-keeping gene [glucose-6-phosphate 1-dehydrogenase (G6PD); *Arahy.XC1VLW* on chromosome 1/*Arahy.74FNJK* on chromosome 11] was worked out for each sample using multiple samples as biological replicates. The fold change across the samples were compared using the t test.

### Results and discussion

On an average, 127,852,977 bisulphite sequencing reads were generated for each sample (Additional file 1: Table S1). As high as 99.96% reads mapped on to the genome of cultivated peanut. Only VG 9514 had relatively low mapped reads, indicating its divergence from the cultivated peanut probably due to the contribution from *A*. *cardenasii.* The number of mapped reads at each DNA methylated site ranged from 1 to 1658 (Additional file 2: Table S2). Among the 11 genotypes, 75,973,928 sites belonged to the category where all the mapped reads (100%) showed cytosine methylation (Additional file 3: Table S3). Similarly, 101,137,805 sites belonged to the category where at least 50% of the mapped reads showed cytosine methylation. The number of sites where less than 50% of the mapped reads showed cytosine methylation was 126,487,183.

On an average, 255,319,879 plausible DNA methylation sites were found among the 11 genotypes, of them 76,886,803 sites showed DNA methylation with 100% reads showing methylation (Table 2). The B sub-genome exhibited higher DNA methylation sites (46,294,063) than the A sub-genome (30,415,166) across the genotypes. A total of 177,574 sites were found in the scaffolds. CHG (where H is A, C or T) region showed the highest methylation sites (30,537,376) regions, followed by CpG (30,356,066) and CHH (15,993,361) regions across the genotypes. This observation is in line with the previous reports [29, 30] that the DNA methylation in plants is found both in CpG and non-CpG (CHG and CHH, where H is A, C or T) contexts in contrast to mammals where DNA methylation occurs predominantly at CpG dinucleotides.

**Table 2:** DNA methylation sites among the peanut genotypes

Sample	CpG				CHG				CHH			
	Total	Methylated	A genome	B genome	Total	Methylated	A genome	B genome	Total	Methylated	A genome	B genome
GPBD 4	31,663,874	29,848,251	11,760,065	18,008,152	40,177,586	29,880,544	11,830,612	17,993,870	178,935,382	17,048,983	6,943,821	10,069,256
VG 9514	32,445,817	30,288,901	11,894,773	18,308,727	41,293,914	31,080,870	12,268,953	18,752,614	183,452,039	14,960,448	6,065,417	8,862,905
ICGV 86855	27,943,111	27,130,352	10,527,102	16,531,496	35,387,700	27,135,584	10,558,531	16,526,472	154,386,730	16,169,355	6,494,227	9,642,136
ICGV 86699	32,688,192	31,020,240	12,102,494	18,834,402	41,574,466	30,412,911	11,916,546	18,438,880	185,674,761	15,272,420	6,163,282	9,076,921
ICGV 99005	34,477,649	32,005,405	12,535,802	19,383,213	43,710,526	31,146,419	12,268,603	18,818,636	195,930,475	15,652,170	6,327,998	9,290,176
TAG 24	33,202,174	31,011,515	12,158,655	18,759,336	42,196,978	31,731,452	12,523,754	19,143,967	189,327,602	15,792,267	6,399,209	9,357,840
TMV 2	28,363,522	26,892,267	10,583,517	16,237,332	35,841,706	27,819,425	10,976,169	16,792,106	154,808,095	14,332,418	5,806,056	8,498,122
JL 24	34,028,898	31,745,351	12,510,873	19,138,621	43,376,521	32,839,584	13,028,058	19,745,221	195,943,632	17,552,832	7,144,759	10,368,667
DER	32,905,294	30,853,653	12,146,023	18,614,509	41,859,554	31,309,671	12,408,504	18,838,577	187,027,809	15,772,260	6,415,665	9,321,787
VL 1	34,335,421	32,427,631	12,747,839	19,592,191	43,825,764	31,789,229	12,559,737	19,169,059	197,280,818	15,795,413	6,401,860	9,360,431
TMV 2-NLM	32,076,131	30,693,163	11,983,385	18,625,032	40,760,931	30,765,451	12,039,163	18,667,382	181,615,597	17,578,402	7,075,377	10,466,654

Among the 11 genotypes, JL 24 and TMV 2 showed the highest (82,137,767) and the lowest methylation sites (69,044,110), respectively (Table 3). Such a natural epigenetic variation was also observed among the different ecotypes of Arabidopsis [31]. Many of the sites were found to be conserved across for DNA methylation across the genotypes of peanut. A total of 5,379,101 sites showed DNA methylation across all the 11 genotypes. The sites showing genotype-specific DNA methylation ranged from 6,575,363 (TMV 2) to 9,190,780 (JL 24) (Table 3).

**Table 3:** DNA methylation pattern among the peanut genotypes

Sample	Total methylated sites	Genic	Exonic	Intronic	Upstream (2 kb)	Downstream (2 kb)	Intergenic	Unique sites	Genes with methylation
GPBD 4	76,777,778	2,542,609	954,981	1,615,686	2,422,857	2,002,208	74,235,169	8,252,512	53,177
VG 9514	76,330,219	2,491,420	926,732	1,591,061	2,353,495	1,938,882	73,838,799	7,557,697	53,344
ICGV 86855	70,435,291	2,236,778	832,360	1,427,817	2,197,445	1,793,923	68,198,513	7,549,158	51,179
ICGV 86699	76,705,571	2,608,306	1,006,455	1,631,016	2,396,086	1,994,004	74,097,265	7,856,020	54,699
ICGV 99005	78,803,994	2,745,542	1,068,392	1,708,747	2,508,886	2,096,784	76,058,452	8,283,176	55,497
TAG 24	78,535,234	2,591,128	972,282	1,647,757	2,459,092	2,043,738	75,944,106	8,207,597	53,838
TMV 2	69,044,110	2,237,309	853,885	1,407,484	2,143,795	1,756,481	66,806,801	6,575,363	51,866
JL 24	82,137,767	2,755,372	1,042,721	1,743,612	2,574,365	2,137,468	79,382,395	9,190,780	54,645
DER	77,935,584	2,555,262	964,170	1,618,932	2,394,118	1,984,129	75,380,322	7,889,130	53,555
VL 1	80,012,273	2,737,311	1,045,365	1,723,338	2,540,239	2,105,390	77,274,962	8,693,213	54,876
TMV 2-NLM	79,037,016	2,675,872	1,018,559	1,687,698	2,533,141	2,103,929	76,361,144	9,031,971	54,463

On an average, inter-genic regions (70,464,637 sites) were more prone for DNA methylation than the genic regions including 2 kb upstream and 2 kb downstream regions (6,422,166 sites) (Table 3). Within the genic regions, the introns (1,590,263) showed a greater number of DNA methylation sites than the exonic regions (971,274). The 2 kb upstream and 2 kb downstream regions had 3,860,629 DNA methylation sites, indicating higher proportion of DNA methylation at the upstream and downstream regions than the gene body region. The distribution of DNA methylation within the genome especially in the promoter and gene body regions is very important as it influences the gene expression [32].

Of the 67,124 genes (31,359 in A genome, 35,110 in B genome and 655 on scaffolds) in peanut, the number of genes showing at least one methylated site ranged from 51,179 (ICGV 86855) to 55,497 (ICGV 99005) (Table 3). Of them, *Arahy.0DU9MH*, a 342,359 bp long gene on chromosome 11, showed the highest number of methylated sites, which ranged from 11,488 (ICGV 86855) to 14,026 (JL 24). Within *Arahy.0DU9MH,* the promoter region had 131 methylated sites, while the gene body (142 in exons and 12,573 in introns) had 12,715 sites. The expression (FPKM) of the 53,740 genes varied widely among the 11 genotypes (Additional file 4: Table S4). *Arahy.0DU9MH* with the highest DNA methylations sites did not show any expression at 21 DAS in the leaves of the 11 genotypes. Fifty genes with wide range of FPKM across the genotypes were selected and checked for the DNA methylation. *Arahy.FHUH7B* on chromosome 10 showing the highest FPKM of 54,951 had a maximum of 102 DNA methylation sites (Additional file 5: Table S5). Many of these genes showed negative association between the number of DNA methylation sites and FPKM among the genotypes.

Fourteen C5-MTase coding genes and ten DNA demethylase coding genes identified in the diploid peanut earlier [21] were analysed for DNA methylation and expression. A considerable variation was observed for methylation across the genes, however, not much variation was observed for methylation across the genotypes (Additional file 6: Table S6). A DME-like A gene *Arahy.R549UJ* (*Aradu.4D5YM*) of 15,531 bp length on chromosome 8 showed the highest number of methylation (as high as 586). Forty-four sites were found in the promoter region, while 542 sites were in the gene body (48 in exon and 494 in intron). This gene did not show any expression at 21 DAS in the leaves of the 11 genotypes.

An attempt was made to enumerate the differentially methylated sites between a parent (TMV 2) and its EMS-derived mutant (TMV 2-NLM). The two genotypes significantly differed for 650 methylation sites, of which 240 and 401 were found in the A and B genome (remaining nine on the scaffolds), respectively. Again, the inter-genic region showed a greater number of DNA methylated sites (605) than the genic regions (45; 23 in exons and 22 in introns). Thirty-seven genes exhibited differential methylation, of which eight showed differential expression (Additional file 7: Table S7a), indicating the influence of EMS mutagenesis on DNA methylation.

In an attempt to identify the differentially DNA methylated sites, foliar disease resistant (GPBD 4, VG 9514, ICGV 86855, ICGV 99005 and ICGV 86699) and susceptible (TAG 24, TMV 2 and JL 24) groups of genotypes were constructed. The common sites within susceptible group were compared with the common sites within the resistant group. In total, 766 sites showed significantly differential DNA methylation. Of these, 331 sites were in the A genome and 433 sites were in the B genome. In total, 731 methylation sites were in the inter-genic regions and 35 were in the genic regions (19 in exons and 16 in introns). Interestingly, four differentially DNA methylated sites (1,001,785, 1,001,813, 1,021,671 and 1,305,680) mapped to the QTL region (for LLS) on A02 and one (134,350,159) mapped to the QTL region (for rust) on A03 [33]. Of these sites, only one (1,305,680) was in a genic (*Arahy.42YDET*) region. However, this gene has not been regarded as a candidate gene for foliar disease resistance [33]. Based on the genomic position of the DNA methylation sites, 25 genes were found to be differentially methylated (*q *≤ 0.01) between resistant and susceptible genotypes. Of these genes, two genes (*Arahy.1XYC2X* on chromosome 01 and *Arahy.00Z2SH* on chromosome 17) coding for senescence-associated protein showed differential expression with resistant genotypes recording higher FPKM values (Additional file 7: Table S7b). It was interesting to note the methylation pattern within *Arahy.1XYC2X* differed between the resistant and susceptible groups, indicating the epialleles at this locus. The candidate genes identified for late leaf spot (four genes) and rust (six genes) resistance in the previous study [33] did not show any DNA methylation, indicating that breeding for foliar disease resistance can depend only on the genetic variation. FPKM values for the transcripts at these loci were *on par* between the resistant and the susceptible genotypes. This was also confirmed by the qRT-PCR where some of these genes showed non-significant fold changes between the two groups (Additional file 8: Table S8).

## Limitations

A detailed genome-wide DNA methylation pattern was reported from the 11 diverse genotypes of peanut for the first time by analyzing the leaf samples collected at 21 DAS. Also, the influence of induced mutagenesis on the pattern of DNA methylation was assessed. Differentially methylated sites were identified between the foliar disease resistant and susceptible genotypes. The genes showing differential methylation and expression were identified. However, these data need to be validated by comparing the samples collected at different stages of growth and development and varying conditions (diseased and normal) to identify the role of DNA methylation, and its influence on gene expression in peanut.

## Supplementary information

**Additional file 1: Table S1.** Features of DNA methylome reads generated in this study.

**Additional file 2: Table S2.** Read depth observed 100% Freq C among the CPG, CHG and CHH regions among the 11 genotypes of peanut.

**Additional file 3: Table S3.** Number of reads with various Freq C values at CPG, CHG and CHH regions among the 11 genotypes of peanut.

**Additional file 4: Table S4.** FPKM values of all the genes of peanut genotypes at seedling stage.

**Additional file 5: Table S5.** DNA methylation pattern for the top 50 genes showing wide range of FPKM values among the peanut genotypes.

**Additional file 6: Table S6.** DNA methylation sites in the DNA methyl transferase and DNA demethylase genes among the peanut genotypes.

**Additional file 7: Table S7a**. Genes showing differential expression between the parental and its mutant progeny genotypes of peanut. **Table S7b.** Genes showing differential expression between the foliar disease resistant and susceptible genotypes of peanut.

**Additional file 8: Table S8.** Real-time PCR analysis for the foliar disease resistance-linked genes among the resistant and the susceptible genotypes of peanut.

## Data Availability

The sequences have been submitted, and the GenBank accession number (DRA007069) has been obtained for the methylome and transcriptome reads of GPBD 4, VG 9514, ICGV 86855, ICGV 86699, ICGV 99005, TAG 24, TMV 2, JL 24, DER, VL 1 and TMV 2-NLM). The genotypes used in this study are either the released varieties or the breeding lines.

## Abbreviations

CHG: C (A, C or T) G
CpG: C phosphate G
CHH: C (A, C or T) (A, C or T)
ELS: early leaf spot
LLS: late leaf spot
FPKM: fragments per kilobase of transcript per million mapped reads
QTL: quantitative trait loci
MET: methyltransferase
CMT: chromomethylase
DRM: DNA methyltransferase
qRT-PCR: quantitative reverse transcription polymerase chain reaction
PEG: polyethylene glycol
cDNA: complementary DNA
SYBR: *N*′,*N*′-dimethyl-*N*-[4-[(*E*)-(3-methyl-1,3-benzothiazol-2-ylidene) methyl]-1-phenylquinolin-1-ium-2-yl]-*N*-propylpropane-1,3-diamine
G6PD: glucose-6-phosphate dehydrogenase
DME: DNA glycosylase DEMETER
QTL: quantitative trait loci
PCR: polymerase chain reaction
