# Supplementary material for: DNA methylation and expression analyses reveal epialleles for the foliar disease resistance genes in peanut (Arachis hypogaea L.)
Source: BMC Res Notes. 2020 Jan 7;13:20. doi: 10.1186/s13104-020-4883-y (PMC6947992; doi:10.1186/s13104-020-4883-y)
Supplement: Supplementary file 1 — Additional file 1: Table S1. Features of DNA methylome reads generated in this study. [file 13104_2020_4883_MOESM1_ESM.docx]

Table S1. Features of DNA methylome reads generated in this study

| **Genotype** | **TOTAL** | **QC PASSED** | **QC PASS**  **(%)** | **ALIGNED** | **ALIGN (%)** | **UNALIGNED** | **UNALIGN**  **(%)** | **Read length (bp)** |
| --- | --- | --- | --- | --- | --- | --- | --- | --- |
| GPBD 4 | 121,288,416 | 120,556,562 | 99.40 | 120,511,468 | 99.96 | 45,094 | 0.04 | 92.2671 |
| VG 9514 | 124,958,064 | 123,901,712 | 99.15 | 123,534,507 | 99.70 | 367,205 | 0.30 | 91.9264 |
| ICGV 86855 | 97,831,782 | 96,972,036 | 99.12 | 96,935,428 | 99.96 | 36,608 | 0.04 | 90.5185 |
| ICGV 86699 | 120,543,650 | 119,574,030 | 99.20 | 119,523,346 | 99.96 | 50,684 | 0.04 | 91.5247 |
| ICGV 99005 | 145,980,230 | 144,153,726 | 98.75 | 144,087,523 | 99.95 | 66,203 | 0.05 | 90.4495 |
| TAG 24 | 134,314,816 | 132,900,982 | 98.95 | 132,841,097 | 99.95 | 59,885 | 0.05 | 90.4283 |
| TMV 2 | 107,226,330 | 106,045,134 | 98.90 | 106,002,797 | 99.96 | 42,337 | 0.04 | 88.8674 |
| JL 24 | 144,948,360 | 143,032,584 | 98.68 | 142,969,196 | 99.96 | 63,388 | 0.04 | 89.5922 |
| DER | 142,594,604 | 140,542,668 | 98.56 | 140,452,132 | 99.94 | 90,536 | 0.06 | 89.2868 |
| VL 1 | 141,146,072 | 139,531,028 | 98.86 | 139,459,074 | 99.95 | 71,954 | 0.05 | 90.3058 |
| TMV 2-NLM | 125,550,428 | 124,013,512 | 98.78 | 123,958,907 | 99.96 | 54,605 | 0.04 | 89.4413 |
