# Supplementary material for: DNA methylation and expression analyses reveal epialleles for the foliar disease resistance genes in peanut (Arachis hypogaea L.)
Source: BMC Res Notes. 2020 Jan 7;13:20. doi: 10.1186/s13104-020-4883-y (PMC6947992; doi:10.1186/s13104-020-4883-y)
Supplement: Supplementary file 2 — Additional file 2: Table S2. Read depth observed 100% Freq C among the CPG, CHG and CHH regions among the 11 genotypes of peanut. [file 13104_2020_4883_MOESM2_ESM.docx]

Table S2. Read depth observed 100% Freq C among the CPG, CHG and CHH regions among the 11 genotypes of peanut

| **Genotype and region** | **Minimum**  **coverage** | **Maximum**  **coverage** | **Average**  **coverage** |
| --- | --- | --- | --- |
| GPBD 4_CHG | 1 | 115 | 2.9 |
| GPBD 4_CHH | 1 | 47 | 1.3 |
| GPBD 4_CpG | 1 | 171 | 3.3 |
| VG 9514_CHG | 1 | 1,658 | 2.9 |
| VG 9514_CHH | 1 | 480 | 1.3 |
| VG 9514_CpG | 1 | 794 | 3.2 |
| ICGV 86855_CHG | 1 | 72 | 2.6 |
| ICGV 86855_CHH | 1 | 55 | 1.3 |
| ICGV 86855_CpG | 1 | 191 | 2.9 |
| ICGV 86699_CHG | 1 | 88 | 2.8 |
| ICGV 86699_CHH | 1 | 44 | 1.3 |
| ICGV 86699_CpG | 1 | 134 | 3.1 |
| ICGV 99005_CHG | 1 | 109 | 3.0 |
| ICGV 99005_CHH | 1 | 43 | 1.3 |
| ICGV 99005_CpG | 1 | 123 | 3.4 |
| TAG 24_CHG | 1 | 89 | 2.9 |
| TAG 24_CHH | 1 | 45 | 1.3 |
| TAG 24_CpG | 1 | 105 | 3.2 |
| TMV 2_CHG | 1 | 85 | 2.7 |
| TMV 2_CHH | 1 | 42 | 1.3 |
| TMV 2_CpG | 1 | 130 | 2.9 |
| JL 24_CHG | 1 | 135 | 3.0 |
| JL 24_CHH | 1 | 52 | 1.3 |
| JL 24_CpG | 1 | 173 | 3.2 |
| DER_CHG | 1 | 274 | 3.0 |
| DER_CHH | 1 | 216 | 1.3 |
| DER_CpG | 1 | 272 | 3.3 |
| VL 1_CHG | 1 | 87 | 2.9 |
| VL 1_CHH | 1 | 63 | 1.3 |
| VL 1_CpG | 1 | 167 | 3.3 |
| TMV 2-NLM_CHG | 1 | 80 | 2.8 |
| TMV 2-NLM _CHH | 1 | 48 | 1.3 |
| TMV 2-NLM _CpG | 1 | 233 | 3.1 |
