# Supplementary material for: DNA methylation and expression analyses reveal epialleles for the foliar disease resistance genes in peanut (Arachis hypogaea L.)
Source: BMC Res Notes. 2020 Jan 7;13:20. doi: 10.1186/s13104-020-4883-y (PMC6947992; doi:10.1186/s13104-020-4883-y)
Supplement: Supplementary file 3 — Additional file 3: Table S3. Number of reads with various Freq C values at CPG, CHG and CHH regions among the 11 genotypes of peanut. [file 13104_2020_4883_MOESM3_ESM.docx]

Table S3. Number of reads with various Freq C values at CPG, CHG and CHH regions among the 11 genotypes of peanut

|  | **CPG** |  |  |  | **CHG** |  |  |  | **CHH** |  |  |  |
| --- | --- | --- | --- | --- | --- | --- | --- | --- | --- | --- | --- | --- |
|  | **Total** | **> 0** | **≥50** | **100** | **Total** | **>0** | **≥50** | **100** | **Total** | **>0** | **≥50** | **100** |
| **GPBD 4** | 35,234,994 | 32,869,711 | 32,706,120 | 29,628,084 | 44,794,590 | 39,148,900 | 37,404,909 | 29,475,739 | 198,512,040 | 56,309,904 | 31,782,400 | 16,658,666 |
| **VG 9514** | 36,092,221 | 33,490,103 | 33,320,136 | 30,056,110 | 46,016,521 | 40,028,939 | 38,298,416 | 30,684,613 | 203,358,963 | 50,759,329 | 27,920,597 | 14,625,629 |
| **ICGV 86855** | 31,057,066 | 29,244,753 | 29,153,906 | 26,972,490 | 39,377,954 | 34,441,615 | 33,118,727 | 26,793,922 | 171,023,543 | 46,767,442 | 28,472,906 | 15,795,246 |
| **ICGV 86699** | 36,337,362 | 33,982,408 | 33,839,294 | 30,842,815 | 46,287,765 | 40,040,524 | 38,259,134 | 30,053,625 | 205,669,999 | 51,449,527 | 28,575,200 | 14,969,433 |
| **ICGV 99005** | 38,321,639 | 35,712,862 | 35,527,509 | 31,763,945 | 48,666,343 | 42,069,998 | 39,986,721 | 30,719,954 | 216,972,983 | 56,764,853 | 30,004,615 | 15,335,417 |
| **TAG 24** | 37,000,976 | 34,360,451 | 34,180,905 | 30,809,797 | 47,071,861 | 41,190,971 | 39,505,705 | 31,365,605 | 209,999,045 | 54,636,583 | 29,881,819 | 15,481,768 |
| **TMV 2** | 31,458,939 | 29,285,049 | 29,163,057 | 26,753,110 | 39,804,403 | 34,810,749 | 33,525,095 | 27,548,740 | 171,048,777 | 42,973,597 | 25,644,474 | 14,050,217 |
| **JL 24** | 37,847,967 | 35,113,537 | 34,918,182 | 31,550,213 | 48,312,989 | 42,182,296 | 40,404,896 | 32,484,522 | 217,034,890 | 59,775,615 | 33,411,580 | 17,196,221 |
| **DER** | 36,629,988 | 34,135,947 | 33,950,109 | 30,618,392 | 46,674,064 | 40,836,329 | 39,015,797 | 30,886,980 | 207,437,120 | 54,949,291 | 29,794,565 | 15,415,229 |
| **VL 1** | 38,227,447 | 35,746,016 | 35,585,737 | 32,211,630 | 48,895,934 | 42,607,891 | 40,700,773 | 31,360,424 | 218,877,078 | 56,450,090 | 30,252,056 | 15,463,965 |
| **TMV 2-NLM** | 35,727,696 | 33,524,998 | 33,400,986 | 30,516,505 | 45,455,342 | 39,820,999 | 38,276,837 | 30,404,977 | 201,462,624 | 55,877,736 | 32,532,696 | 17,219,221 |
| **Mean** | 35,812,390 | 33,405,985 | 33,249,631 | 30,156,645 | 45,577,979 | 39,743,565 | 38,045,183 | 30,161,736 | 201,945,187 | 53,337,633 | 29,842,992 | 15,655,547 |
